# Distance Measurement and Data Analysis for Civil Aviation at 1000 Frames per Second Using Single-Photon Detection Technology

**DOI:** 10.3390/s25133918

**Published:** 2025-06-24

**Authors:** Yiming Shan, Xinyu Pang, Huan Wang, Jitong Zhao, Shuai Yang, Yunlong Li, Guicheng Xu, Lihua Cai, Zhenyu Liu, Xiaoming Wang, Yi Yu

**Affiliations:** 1 Changchun Institute of Optics, Fine Mechanics and Physics, Chinese Academy of Sciences, Changchun 130033, China; shanyiming23@mails.ucas.ac.cn (Y.S.); pangxinyu@ciomp.ac.cn (X.P.); zhaojitong@ciomp.ac.cn (J.Z.); xuguicheng24@mails.ucas.ac.cn (G.X.); cailihua@ciomp.ac.cn (L.C.); liuzy@ciomp.ac.cn (Z.L.); 2 Changchun Institute of Optics, Fine Mechanics and Physics, Changchun 130033, China; yangshuai65535@163.com (S.Y.); liyunlong@ciomp.ac.cn (Y.L.); wangxiaoming@ciomp.ac.cn (X.W.)

**Keywords:** TCSPC technology, high-speed target measurement, laser radar

## Abstract

During high-speed maneuvers, aircraft experience rapid distance changes, necessitating high-frame-rate ranging for accurate characterization. However, existing optical ranging technologies often lack simplicity, affordability, and sufficient frame rates. While dual-station triangulation enables high-frame-rate distance calculation via geometry, it suffers from complex and costly deployment. Conventional laser rangefinders are limited by low repetition rates. Single-photon ranging, using high-frequency low-energy pulses and detecting single reflected photons, offers a promising alternative. This study presents a kilohertz-level single-photon ranging system validated through civil aviation field tests. At 1000 Hz, relative distance, velocity, and acceleration were successfully captured. Simulating lower frame rates (100 Hz, 50 Hz, 10 Hz) via misalignment merging revealed standard deviations of 0.1661 m, 0.2361 m, and 0.2683 m, respectively, indicating that higher frame rates enhance distance measurement reproducibility. Error analysis against the 1000 Hz baseline further confirms that high-frame-rate ranging improves precision when monitoring high-speed maneuvers.

## 1. Introduction

High-speed maneuvering is an important performance characteristic of aircraft, missiles, and other flying vehicles [1]. Some critical maneuvers are completed in a very short period, which requires optical measurement devices to have high-frame-rate, multi-dimensional detection capabilities to fully capture the high-speed maneuvering process. Currently, optical measurement devices mainly use medium/long-wave infrared cameras to capture two-dimensional images of the maneuvering process, with frame rates reaching up to approximately 400 Hz [2,3], which can meet the imaging requirements of most high-speed maneuvers in two dimensions. However, two-dimensional images lack distance information and cannot fully characterize the maneuvering process of the aircraft. By controlling multiple optical measurement devices to simultaneously observe the target, the distance to the target can be calculated through geometric relationships. This method is known as “dual-station intersection ranging” [4,5,6]. In principle, the frame rate of intersection ranging can reach the imaging frame rate of the optical measurement devices. However, the “dual-station intersection” system is complex, and its inherent geometric structure limits the field of view. Additionally, the high cost of deploying multiple stations makes this method impractical, thus highlighting the need for alternative high-frame-rate ranging methods.

Laser ranging technology can measure the distance traveled by flying vehicles [7,8,9]. However, atmospheric scattering, absorption, and other effects significantly attenuate the laser, while conventional detectors require a specific response threshold [10] (typically 1 nanojoule, equivalent to 1 billion photons). This leads to high energy demands for laser pulses when detecting distant targets, which, in turn, reduces the laser’s emission frequency [11]. Consequently, the ranging frame rates are also lower. The frame rates of conventional laser rangefinders typically range from 20 Hz to no more than 50 Hz, often requiring frame interpolation [12,13,14] to match those of medium/long-wave infrared imaging. As a result, accurately depicting high-speed maneuvers becomes challenging [15,16,17].

In recent years, single-photon ranging technology has developed rapidly [18,19], with single-photon detectors configured to have response thresholds as low as a single photon. When emitting relatively low-energy laser pulses toward the target, a small number of signal photons can still return and trigger the detector, even after long-distance transmission and atmospheric attenuation. Consequently, single-photon ranging systems no longer rely on high-pulse-energy lasers; instead, low-pulse-energy lasers suffice for long-distance ranging [20,21]. Moreover, low-pulse-energy lasers can operate at higher repetition rates, thereby increasing the frame rate of single-photon ranging and offering the potential for high-frame-rate distance measurement.

Current research on single-photon ranging predominantly focuses on extending the detection range [22], suppressing noise [23], scanning imaging [24], and intelligent image recognition [25]. However, limited attention has been paid to enhancing frame rates for ranging applications involving high-speed targets. Achieving high-frame-rate ranging necessitates each frame being acquired within a very short integration time, rendering long-integration-based signal enhancement strategies ineffective for reliable signal extraction. This imposes stringent requirements on the optical noise suppression capabilities and denoising algorithms of the ranging system. To meet these demands, we developed a high-efficiency photon detection and acquisition system capable of preliminarily identifying photon signals without relying on extended integration times. Building on this foundation, we adopted a density-based clustering algorithm for denoising, aligned with the temporal and statistical properties of high-frame-rate photon detection data, thereby enabling the precise extraction of weak photonic signals. We successfully performed the thousand-frame-per-sequence tracking and ranging of civil aircraft and estimated their derived radial velocity and acceleration. The standard deviations of ranging measurements at different frame rates (100 Hz, 50 Hz, and 10 Hz) were quantified as 0.1661 m, 0.2361 m, and 0.2683 m, respectively. The analysis indicates that higher frame rates correspond to smaller standard deviations, indicating enhanced measurement reproducibility and reduced signal variance—thereby improving both the “credibility” and “reliability” of the data. We further performed regression analysis on radial kinematic parameters under various frame rate conditions (100 Hz, 50 Hz, 10 Hz), and demonstrated that increasing frame rates substantially reduced regression residuals. These results confirm that the proposed system is suitable for real-time monitoring of high-mobility aerial platforms, providing a viable solution for detecting high-speed maneuvers of airborne vehicles.

## 2. Principle of Single-Photon Laser Radar Distance Measurement

**Figure 1:**
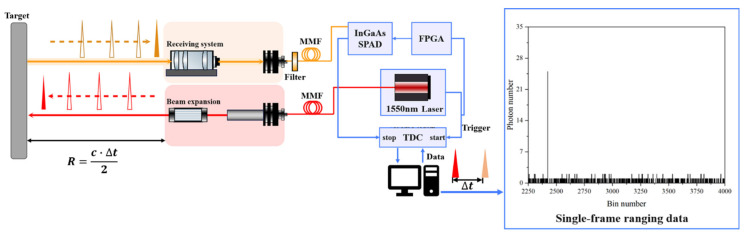
Schematic of time-of-flight single-photon ranging system.

A pulsed laser initiates a start timing signal, delivered to a time-to-digital converter (TDC), and simultaneously emits a laser pulse, which is expanded via a beam expander and projected onto a distant target. Photons scattered from the target are detected by a single-photon detector, which generates an electrical pulse, serving as the stop signal. Both the start and stop signals are fed into a time-correlated single-photon counting (TCSPC) system, which records the time of flight (TOF) of the detected photons along with the associated pulse repetition sequence. After repeated accumulation over multiple cycles, a time-correlated single-photon counting histogram is generated, depicting the temporal distribution of photon arrivals, as schematically illustrated in Figure 1.

The cumulative acquisition of photon time-of-flight and pulse repetition sequence data yields a time-correlated single-photon counting histogram. Peak detection [26], threshold detection [27], and centroid detection [28] are subsequently employed to extract the photon pulse’s time of arrival, denoted as $t_{top}$. This temporal parameter enables accurate distance estimation based on the time-of-flight (TOF) principle.

(1) $$R=\frac{c\cdot t_{top}}{2}$$

In this equation, R denotes the target distance, and c denotes the speed of light.

The extremely short per-frame integration time in high-frame-rate single-photon detection precludes us from achieving a high signal-to-noise ratio (*SNR*) by simply extending the integration time, as is common in conventional laser ranging systems. To compensate, the system must exhibit excellent optical noise suppression and incorporate robust data denoising algorithms. To address this challenge, a high-efficiency noise-suppressing photon reception system was developed in this study. It employed an QCD600B-S InGaAs/InP single-photon detector (China Electronics Technology Group Corporation, Chongqing, China), featuring a 45 mm photon reception aperture, a field of view of approximately 200 μrad, and a spectral bandwidth of 2 nm. The detection efficiency is 25%, the dark count rate is 2 kHz, the dead time is set to 0.9 s, and the operating temperature is at 25 °C. The transmitter emits laser pulses at a wavelength of 1550 nm with a pulse width of 1 ns. The time bin width is 10 ns, and the data transmission rate reaches 8 MTags/s. The ranging system is integrated with a theodolite-based tracking platform to enable autonomous target tracking.

Commercial aircraft within a 20 km range are employed in this study as ranging targets. These aircraft constitute extended targets whose dimensions greatly exceed the receiver’s field of view. Thus, all portions of the target within the field of view contribute to the reflected echo signal. As single-photon laser ranging relies on photon counting, the number of echo photons collected by the receiving optics can be expressed as follows [29]:

(2) $$N_{r}=\rho \eta_{t}\eta_{r}T^{2}\cos\theta_{t}\frac{A_{r}}{\pi R^{2}}\frac{\lambda }{hc}E_{t}$$

In this context, ρ represents the target reflectivity (~0.2), $\eta_{t}$ is the laser radar transmission optical efficiency (~62%), $\eta_{r}$ is the reception optical system efficiency (~60%), T is the one-way transmission transmittance of the laser through the atmosphere (~0.6933), $\theta_{t}$ is the angle between the laser emission axis and the vertical direction of the target (set to 0°), $A_{r}$ is the effective area of the receiving optical system (~$45\;{\mathrm{mm}}^{2}$), R is the operational range (~20 km), h is Planck’s constant, set as $6.626\times 10^{-34}\;J\cdot s$, λ is the laser wavelength (1550 nm), *c* is the speed of light, set as $2.9979\times 10^{8}\;m/s$, and $E_{t}$ is the laser single-pulse emission energy (~45 μJ). Based on these parameters, the average number of echo photons received by the system is approximately 19.22 counts per second (cps).

In single-photon light detection and ranging (LiDAR) systems, the detector not only captures photons that are diffusely reflected from the target but also unwanted signals, including background noise and dark counts resulting from the detector’s intrinsic dark current. The primary sources of background noise include solar background noise $P_{sun}$, blackbody radiation from the target $P_{rad}$, and laser backscatter $P_{laser}$. Specifically, in the spectral range from visible to near-infrared, blackbody radiation is significantly weaker than solar background noise and can therefore be neglected. Likewise, the contribution of laser backscatter is typically minimal. As a result, ambient solar light during the daytime constitutes the dominant source of interference. The average number of background photons incident on the detector under daylight conditions is given as follows [30]:

(3) $$N_{b}=\frac{\pi }{4}n_{\lambda }L_{\lambda }\theta_{r}^{2}A_{r}\eta_{r}\Delta \lambda$$

In this context, $n_{\lambda }$ represents the average photon count per watt at the corresponding wavelength ($7.8\times 10^{18}\mathrm{counts}/s\;@1550\mathrm{nm}$), $L_{\lambda }$ is the background radiation brightness ($50\mathrm{mW}/m^{2}\cdot \mathrm{Sr}\cdot \mathrm{nm}$), $\theta_{r}$ is the system’s field-of-view angle (~200 μrad), and Δλ is the bandwidth of the optical filter system (2 nm). Upon calculation, the average background photon count incident on the detector is approximately 3.26 cps. The theoretical *SNR* of the ranging system is as follows: $SNR_{t}=\frac{N_{r}}{N_{b}}=5.8957\mathrm{dB}$.

In addition, the detection frame rate of this system can be calculated using the following formula:

(4) $$F_{frame}=\frac{PRF}{M}=\frac{PRF\cdot \eta \cdot N_{r}}{k}$$

where PRF refers to the corresponding repetition frequency of the pulse laser (~50 kHz), M represents the effective pulse count, η denotes the detection efficiency of the detector (~25%), and k is the ideal signal photon count under the allowable *SNR* condition (~20). $N_{r}$ refers to the photon count of the above-mentioned single pulse echo. Calculations indicate that the theoretical ranging frequency of this system can reach 1.2 kHz. Since the target selected in this paper is a civil aviation target, a frame frequency of 1000 Hz is sufficient to complete the ranging task. To further enhance the theoretical ranging frame frequency, the following solutions can be implemented:

1. Dead Time Mitigation: By implementing active quenching circuits and parallel readout architectures, the SPAD reset time can be reduced.
2. Algorithm-Hardware Co-design: Replace the standard histogram-based processing with sparse event-driven algorithms.

However, the aforementioned improvement plan is currently only at the theoretical conception stage and requires further experimental validation.

## 3. Civil Aviation Thousand Frame Distance Measurement

### 3.1. Distance Measurement Experiment

In long-range ranging applications, the system’s effective measurement range is constrained by the maximum unambiguous distance, which is determined by the laser pulse repetition period (T). When the pulse repetition frequency is insufficient, the system fails to resolve the true target range due to a phenomenon known as range ambiguity [31]. To address this issue, this study adopts a triple-frequency laser pulse emission strategy with repetition frequencies of 50 kHz, 49.5 kHz, and 49 kHz. The true target distance can be determined by solving a system of congruence equations [32], with detailed theoretical derivations provided in the Supplementary Materials.

(5) $$R_{real}=\frac{c\left(m_{1}T_{1}+t_{1}\right)}{2}=\frac{c\left(m_{2}T_{2}+t_{2}\right)}{2}=\frac{c\left(m_{3}T_{3}+t_{3}\right)}{2}$$

In Equation (4), m_1_, m_2_, and m_3_ denote the number of periods corresponding to three ranging signals with different repetition frequencies and are the unknowns to be determined. T_1_, T_2_, and T_3_ represent the pulse periods corresponding to the three repetition frequencies and are known parameters. t_1_, t_2_, and t_3_ correspond to the bin positions of the ranging peaks under each of the three repetition frequencies in the raw data, which are also known. As evident from the equation, in this system, it is necessary to combine three consecutive single-frame ranging datasets obtained under different repetition frequencies to derive a single target distance measurement.

**Figure 2:**
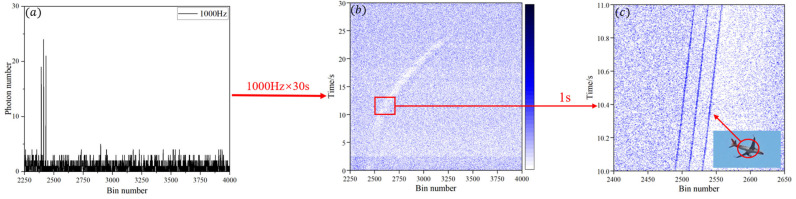
Original data of civil aviation kilo-frame tracking range. (**a**) Photon accumulation diagram of triple-frequency single-frame ranging. (**b**) Original data of civil aviation kilo-frame tracking range with continuous measurement time of 30 s. (**c**) Magnified view of local section of civil aviation kilo-frame ranging data.

Figure 2a shows the single-frame ranging data at three different repetition frequencies. The x-axis represents the time bins where the photons are located, and the y-axis represents the photon count during the integration time of a single frame. The integration time for each repetition frequency is 1 ms, and the single-frame ranging data includes three target peaks corresponding to the three repetition frequencies.

Building on Figure 2a, the photon count is represented by the brightness of the color blocks, and 1000 frames of ranging data are arranged according to the detection time. The single-frame ranging data is displayed as vertical lines, resulting in the “Civil Aviation 1000-frame ranging plot,” shown in Figure 2b. In this figure, the y-axis represents the ranging duration, and the x-axis still represents the time bins where the photons are located. At this point, the original 1000-frame ranging data displays three ranging signal lines, corresponding to the three repetition frequencies.

Moreover, it is observed that random noise signals are distributed outside the signal lines. The average noise count rate in a single frame is approximately 4.33 cps, while the average signal photon count rate is about 21 cps. The *SNR* is 4.85 dB, with an error of 17.64% compared to the theoretical *SNR*. This *SNR* data indicates that the optical system of this ranging device has excellent noise suppression capabilities, allowing preliminary signal recognition by the naked eye before any filtering algorithm is applied. However, the further processing of the ranging data would require the application of specific noise-filtering algorithms to the raw data.

### 3.2. Noise-Filtering Algorithm

The raw distance measurement data from 1000 frames shown in Figure 2 indicates that, due to the extremely short integration time of high-frame-rate distance measurements, the signal strength may be comparable to the noise level. Therefore, noise must be filtered out before further analysis. Upon observation, it is evident that the noise in this graph is much less dense than the distance measurement signal line, which exhibits a linear structure. Based on this, a density-based clustering denoising method [33,34] is chosen for noise filtering in this study. The flowchart of the denoising method is shown in Supplementary Figure S2. First, a fixed sliding window size is set (a rectangular window with a row length of 1 and a column length of 6). Density thresholds are applied in both the row and column directions. The density of non-zero points in the sliding window is compared with the preset threshold; values above the threshold are classified as signals, while those below the threshold are classified as noise. The *SNR* of the denoised data is then calculated under the specified density threshold condition. Once the *SNR* meets the required criteria, the denoised data is output and visualized. The *SNR* is defined as the ratio of the maximum signal count to the root mean square (RMS) of the noise, reflecting the strength of the signal relative to the noise in the measurement.

**Figure 3:**
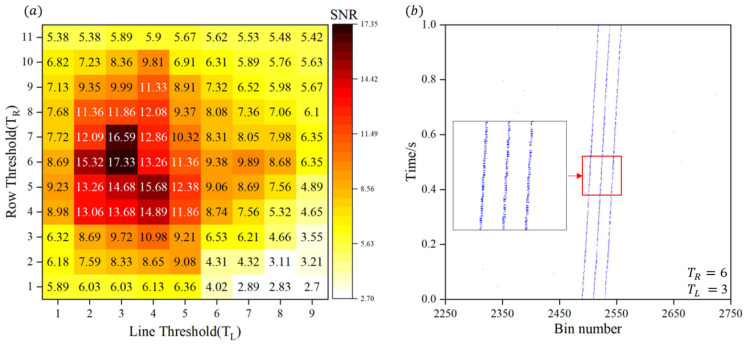
(**a**) Impact of density threshold settings in row and column directions on denoising performance. Low threshold fails to sufficiently suppress noise, leading to reduced *SNR*, while excessively high threshold may result in unintended suppression of valid signals. (**b**) Illustration of optimal denoising performance ($T_{R}=6$, $T_{L}=3$).

The impact of the chosen density threshold on denoising performance is shown in Figure 3a. Based on the average *SNR* curve, the optimal density thresholds are $T_{R}=6$ and $T_{L}=3$. The optimal denoising result is presented in Figure 3b. Compared to the original data, a substantial amount of noise points is removed. After denoising, the *SNR* is 17.33 dB, the target echo signal is much clearer, and the edges are smoother, effectively preserving the key data characteristics of the signal.

In practical engineering survey processes, due to the significant dynamic nature of sunlight noise that changes over time, the *SNR* of raw distance measurement data collected at different time intervals also varies accordingly. This variation places higher demands on subsequent data processing. In this study, a noise-filtering algorithm is proposed based on density clustering. The core idea is to adjust the thresholds for density clustering in both row and column directions to identify and eliminate noise points, thereby improving the quality of the data. However, the effectiveness of the filtering largely depends on whether the selected density thresholds are appropriate.

**Figure 4:**
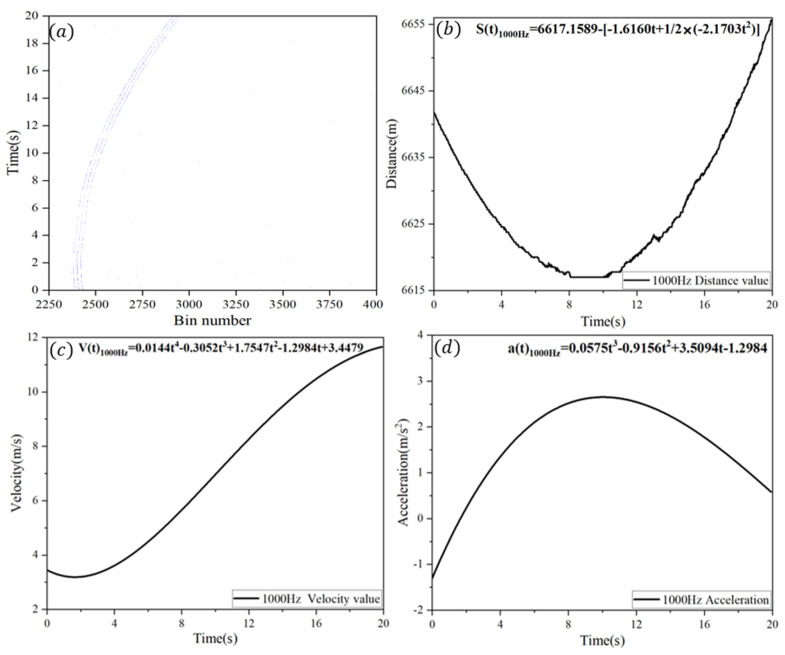
Fitting of civil aviation motion parameters. (**a**) Curve of original ranging data for civil aviation over 20 s measurement interval. (**b**) Fitted curve of civil aviation radial distance. (**c**) Fitted curve of civil aviation radial velocity. (**d**) Fitted curve of civil aviation radial acceleration.

Therefore, this study systematically analyzes the changes in single-frame *SNR* before and after filtering for the raw distance measurement data collected at different time intervals throughout the day. Based on this analysis, the optimal density threshold for achieving the best filtering effect for each time period is determined. The results of the analysis are summarized in Figure 4, which lists the original *SNR*, the post-filtering *SNR*, and the corresponding optimal density parameters for each time period. The *SNR* in Table 1 is the single-frame *SNR* at the time of the test; this description will not be repeated in the future, and only the *SNR* is used.

Table 1 shows hourly variations in *SNR* from 07:30 to 19:30 (local time in Changchun, China), including measurements of raw and denoised *SNR*, along with the corresponding denoising parameters ($T_{R}$ and $T_{L}$). Several key patterns emerge: overall, the raw *SNR* declines throughout the morning, gradually recovers in the afternoon, and reaches a peak in the evening. It begins at 17.31 dB at 07:30, falls to a minimum of 4.66 dB at 11:30, and climbs to a maximum of 22.33 dB by 19:30. This pronounced “V”-shaped trend corresponds to the temporal dynamics of the solar zenith angle (i.e., the angle between the sun and the zenith). At 07:30 and 19:30, when the solar zenith angle is large and the sun is near the horizon, sky brightness is low and background radiation is weak. Under these conditions, ambient light is reduced and causes less radiative interference with the observed signals, allowing for a relatively high *SNR* despite the presence of noise in the raw data. In contrast, during midday—particularly between 11:30 and 13:30—when the solar zenith angle reaches its minimum and the sun is nearly overhead, intensified ground and atmospheric reflections and scattering result in substantial high-frequency background noise. This coincides with the lowest observed *SNR* at 11:30.

After applying noise reduction, the *SNR* improved significantly, particularly during the morning and evening periods. Notably, at 07:30, the *SNR* increased to 23.65 dB, and at 19:30 it rose to 27.69 dB. This suggests that when background illumination is weak and environmental noise is minimal, the noise reduction algorithm operates efficiently, with the selected parameters $T_{R}$ and $T_{L}$ notably enhancing signal extraction. However, at the lowest *SNR* point, 11:30, even with the maximum value of $T_{R}\;$ = 15, the *SNR* only increased to 6.31 dB, indicating limited improvement. This highlights a critical limitation: when background noise is excessive or the signal is dominated by strong background illumination, traditional noise reduction algorithms struggle to yield satisfactory results.

Regarding the noise reduction parameter configuration, $T_{R}$ demonstrates a progressive decline, peaking at 15 at 11:30, suggesting that the system adopted a heightened suppression strategy during periods of severe noise. In contrast, $T_{L}$ values were largely fixed at 2 or 3. This can be attributed to the longitudinal nature of the system’s distance measurement data and the corresponding orientation of the rectangular noise-filtering window employed by the algorithm—namely, its row-wise dimension exceeds its column-wise dimension. As a result, the point density threshold in the row direction is substantially lower than that in the column direction, reflecting the anisotropic design of the denoising algorithm.

Overall, this set of data not only reveals the intra-day changes in the *SNR*, but also makes the explanation of *SNR* variation more scientific and rational by incorporating the physical aspects of solar angle and daylight background noise. At the same time, the adaptive capability of the noise reduction algorithm in dynamic noise environments deserves attention: significant improvements can be achieved during low-background-light periods, while the noise reduction effect should be cautiously evaluated under strong background interference. In other words, the recoverability of the signal is ultimately determined by the tension between the quality of the original signal and the noise background, with parameter adjustments being limited to optimization in relation to this physical boundary. This figure also provides an actionable reference for selecting density thresholds in practical applications. This article currently analyzes civil aviation ranging data of approximately 20 km. Further research will involve the extensive testing of various target types under different distances and environmental conditions. In future measurement tasks, experimenters can use the data from Table 1 to select appropriate density thresholds based on measurement time, achieving more stable and reliable noise suppression effects.

## 4. Analysis of Distance Measurement Results

### 4.1. Data Processing

To obtain the frame-by-frame radial distance of the civil aircraft relative to the ranging device, the denoised ranging data (as shown in Figure 4a, where only 20 s of data are displayed) were processed frame by frame and inserted into Equation (4). By arranging the radial distance data of each frame sequentially over time, the radial distance curve of the civil aircraft over the entire measurement period was obtained, as illustrated in Figure 4b. Given that the motion of the civil aircraft can be considered a non-uniformly accelerated movement, Equation (5) was employed to fit the radial distance curve, and the fitting results are also shown in Figure 4b.

(6) $$S\left(t\right)=S_{0}-\left[V_{0}\left(t\right)\times t+\frac{1}{2}a\left(t\right)\times t^{2}\right]$$

In this equation, $S_{0}$ and $S\left(t\right)$ denote the relative distances between the single-photon ranging system and the target at the initial moment and at time t, respectively. $V_{0}\left(t\right)$ and $a\left(t\right)$ represent the time-varying relative velocity and acceleration of the target. As civil aircraft experience non-uniform acceleration relative to the ranging device, both $V_{0}\left(t\right)$ and $a\left(t\right)$ are modeled as time-dependent functions. In this study, the target’s radial velocity and acceleration curves were derived by fitting the radial distance data using a high-order Taylor series and computing their derivatives, as illustrated in Figure 4c,d.

### 4.2. The Impact of Frame Rate on Distance Measurement Precision

This study employed commercial aircraft as ranging targets. Due to slight variations in flight paths of the same aircraft on different days, each flight afforded a single valid ranging opportunity per day. To assess the impact of the frame rate on ranging performance, initial experiments were conducted at a high frame rate. Subsequently, lower-frame-rate results were simulated by aggregating data, allowing for comparative analysis of ranging performance at both high and low frame rates.

**Figure 5:**
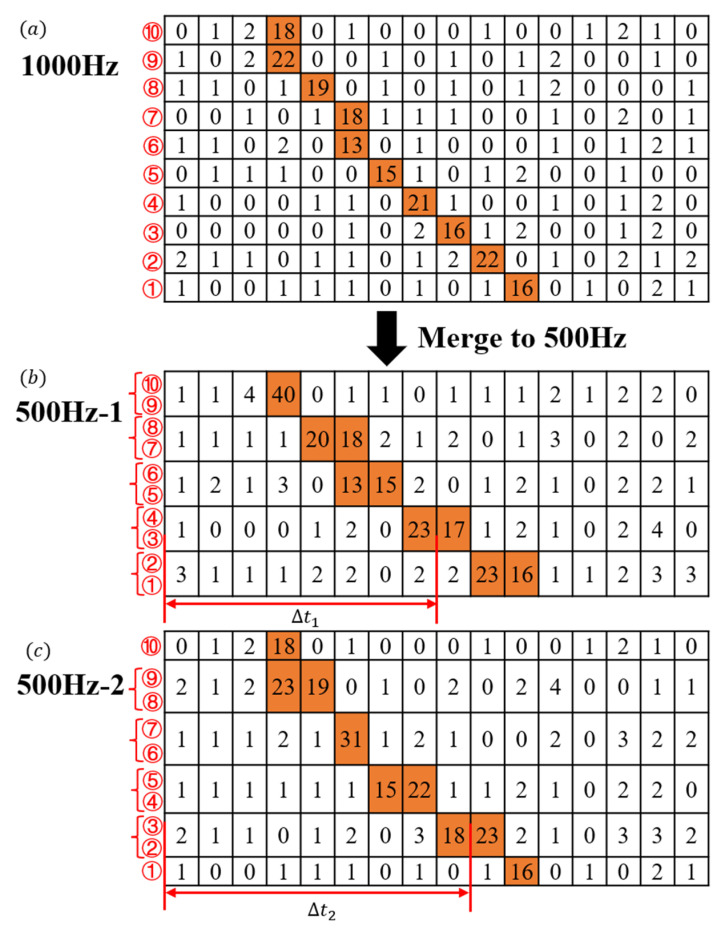
Schematic of misaligned data merging: (**a**) 1000 Hz distance measurement data comprising 10 frames; (**b**) 500 Hz data merged starting from first frame; (**c**) 500 Hz data merged starting from second frame.

Specifically, ranging was performed with a temporal resolution of 10 ns and a frame rate of 1000 Hz. The ranging results are shown in Figure 5a, with a representative set of ten frames displayed. To simulate a frame rate of 500 Hz under the same 10 ns resolution, data from every two consecutive 1000 Hz frames were aggregated into one; the detailed aggregation process is depicted in Figure 5. Data corresponding to even lower frame rates (e.g., 100 Hz, 50 Hz, 10 Hz) can be obtained using the same aggregation method.

Of particular importance is the observation that the order in which high-frame-rate data frames are aggregated has a substantial impact on the resulting low-frame-rate ranging measurements. As depicted in Figure 5, the 500 Hz-1 dataset, generated by integrating the 1st and 2nd frames, is shown in Figure 5b, whereas the 500 Hz-2 dataset, obtained by integrating the 2nd and 3rd frames, is illustrated in Figure 5c. An offset is evident between ∆t_1_ and ∆t_2_, reflecting variations in the time of flight associated with the target signal. This, in turn, results in subtle discrepancies in the final ranging measurements. Nevertheless, these differences originate from authentic ranging data and underscore the measurement errors introduced by reduced frame rates. This finding highlights the importance of frame synchronization in precision-sensitive ranging applications.

To evaluate the range measurement error introduced by lower frame rates, this study adopts the standard deviation method. Based on the aforementioned temporal misalignment-based fusion approach, datasets were constructed after sampling at 100 Hz, 50 Hz, and 10 Hz. Each frame rate includes five distinct datasets generated through different temporal fusion sequences. The single-frame standard deviation s under each frame rate is estimated using the following formula:

(7) $$s=\sqrt{\frac{1}{N-1}\sum_{i=1}^{N}{\left(x_{i}-\hat{x}\right)}^{2}}$$

In this equation, N denotes the number of distance measurements, where $x_{i}$ represents the distance from the i-th measurement and $\hat{x}$ is the average of the five measurements.

**Figure 6:**
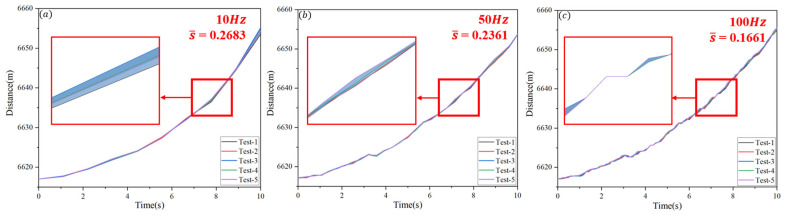
Range measurement data collected over duration of 10 s at different frame rates, along with corresponding average standard deviation. (**a**) Distance measurement data and its local magnification when the ranging frame frequency is 10 Hz, the standard deviation is 0.2683. (**b**) Distance measurement data and its local magnification when the ranging frame frequency is 50 Hz, the standard deviation is 0.2361. (**c**) Distance measurement data and its local magnification when the ranging frame frequency is 50 Hz, the standard deviation is 0.1661.

Working according to Equation (6), the standard deviation of single-frame measurement data collected over 10 s was calculated for different ranging frame frequencies (100 Hz, 50 Hz, and 10 Hz). The average standard deviation, denoted as $\bar{s}$, was then obtained by averaging all individual frame standard deviations. Figure 6 presents the results. As the ranging frame frequency increased from 10 Hz to 50 Hz and 100 Hz, the deviation between the five datasets gradually decreased. The average standard deviations decreased from 0.2683 m and 0.2361 m to 0.1661 m, respectively.

In a distance measurement system, the true position of the target is a fixed value, while each measurement result has a certain degree of error. The smaller the standard deviation of the measurement error, the more stable and accurate the results will be, meaning the system output is closer to the true value. Such low-amplitude changes indicate that the system can provide more reliable and accurate distance data at high frame rates by reducing the impact of temporal measurement variability, thus enhancing the overall reproducibility and reliability of the system.

This study uses civil aircraft as the target, dynamically varying velocity and acceleration relative to the ranging device. Based on this characteristic, the errors in radial distance, radial velocity, and radial acceleration were analyzed under different frame rates (100 Hz, 50 Hz, and 10 Hz) in order to investigate the impact of frame rate on the detection of variable-speed targets using multidimensional information.

**Figure 7:**
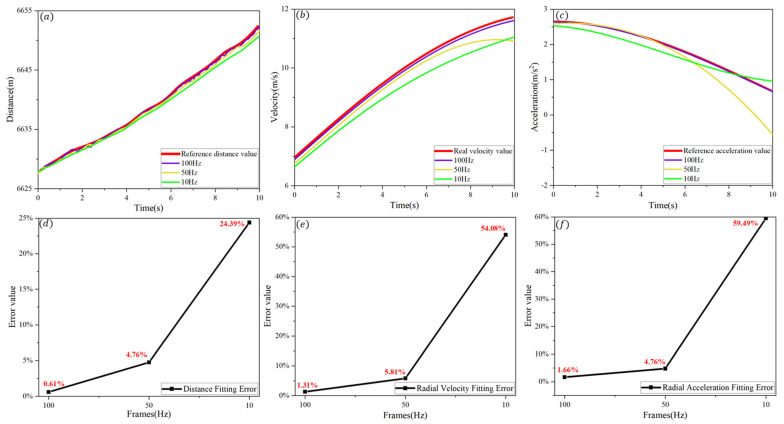
Error between fitted ranging data and reference values at different ranging frame frequencies. (**a**) Distance data for different frame rates. (**b**) Radial velocity data at different frame rates. (**c**) Radial acceleration data at different frame rates. (**d**) Distance fitting error data under different ranging frame frequency conditions. (**e**) Radial velocity fitting error data under different ranging frame frequency conditions. (**f**) Data on fitting errors of radial acceleration under different ranging frame frequency conditions.

The analysis of the standard deviation of ranging measurements reveals a decreasing trend in average error as frame rate increases, indicating improved reliability and stability at higher frame rates. Based on these findings, it is reasonable to infer that ranging data acquired at 1000 Hz exhibit superior stability and reliability. Consequently, the 1000 Hz measurements were adopted as the reference when calculating errors at lower frame rates. The merged data at 10 Hz, 50 Hz, and 100 Hz were each subjected to curve fitting and subsequent error analysis, as presented in Figure 7. The figure clearly demonstrates that fitting errors for all three kinematic parameters increase significantly with a decreasing frame rate. Specifically, the fitting error for radial distance increased from 0.61% and 4.76% to 24.39%; the error for radial velocity rose from 1.31% and 5.81% to 54.08%; and the error for radial acceleration increased from 1.66% and 4.76% to 59.49%. Regarding the aforementioned error data, the error obtained at a 50 Hz ranging frame rate is still within the marginally acceptable range, while the error obtained at a 10 Hz ranging frame rate is unacceptable.

Taken together, these findings suggest that higher frame rates result in reduced measurement errors in ranging data and their derived kinematic parameters, thereby enhancing overall ranging precision.

## 5. Conclusions

To meet the demands of the high-frame-rate detection of rapidly moving objects, this study developed a high-frame-rate single-photon laser ranging system. A thousand-frame laser tracking and ranging experiment was conducted on a civil aviation object. To denoise the extensive dataset, a density-based clustering algorithm was employed. This was chosen based on the distribution characteristics of the thousand-frame data. Using this approach, the relative distance, radial velocity, and radial acceleration of the object were estimated through polynomial fitting. The standard deviations for different ranging frame rates (100 Hz, 50 Hz, and 10 Hz) were analyzed, yielding values of 0.1661 m, 0.2361 m, and 0.2683 m, respectively. The results indicate that increased frame rates led to smaller average standard deviations, thus enhancing ranging precision. Additionally, the fitted values for radial distance, velocity, and acceleration under varying frame rate conditions were compared to a reference 1000 Hz dataset. The analysis showed that higher frame rates led to reduced fitting errors. These findings demonstrate the high precision of high-frame-rate single-photon laser ranging when capturing rapidly maneuvering objects. The proposed system shows significant potential in monitoring high-speed maneuvers, providing a novel approach for tracking the dynamic behavior of fast-moving objects.

## Figures and Tables

**Table 1:** Comparison of denoising parameters and performance across diurnal time points.

Detection Time	Original Data SNR (dB)	Target Location		Sun Location		Noise Reduction Parameters		SNR of Filtered Data (dB)
		Azimuth	Elevation	Azimuth	Elevation	$T_{R}$	$T_{L}$	
07:30	17.31	97°02′26″	34°07′12″	79°59′24″	19°08′24″	9	3	23.65
08:30	16.56	107°19′30″	41°02′15″	96°46′40″	29°46′48″	8	3	21.77
09:30	13.23	104°12′03″	47°06′26″	116°7′48″	39°11′24″	10	3	21.86
10:30	9.84	107°22′12″	53°15′11″	134°24′26″	47°42′06″	9	2	10.33
11:30	4.66	89°01′18″	41°15′38″	153°37′48″	53°15′36″	15	4	6.31
12:30	5.81	101°27′46″	56°12′36″	173°06′02″	56°21′26″	12	3	8.62
13:30	6.31	109°10′48″	54°39′36″	192°15′36″	56°53′24″	9	2	10.68
14:30	5.21	107°06′15″	49°12′36″	221°07′02″	56°04′48″	7	3	16.31
15:30	8.67	105°31′36″	53°22′48″	227°43′48″	52°09′36″	9	3	13.86
16:30	13.83	104°15′08″	46°02′21″	247°25′12″	47°01′12″	8	2	19.86
17:30	21.92	112°25′17″	40°18′13″	266°48′21″	40°46′36″	7	2	26.32
18:30	21.63	102°18′35″	47°38′15″	286°47′24″	29°29′24″	6	2	28.36
19:30	22.33	105°33′06″	52°48′06″	305°19′55″	17°05′31″	5	3	27.69

## Data Availability

Data underlying the results presented in this paper are not publicly available at this time but may be obtained from the authors upon reasonable request.

## Supplementary Materials

The following supporting information can be downloaded at: https://www.mdpi.com/article/10.3390/s25133918/s1, Figure S1: (a) The process of the formation of blurred distances. (b) Principle of Multi-frequency Technology. Figure S2: Flowchart of the denoising algorithm based on density clustering.
