# Supplementary material for: Distance Measurement and Data Analysis for Civil Aviation at 1000 Frames per Second Using Single-Photon Detection Technology
Source: Sensors (Basel). 2025 Jun 24;25(13):3918. doi: 10.3390/s25133918 (PMC12251673; doi:10.3390/s25133918)
Supplement: Supplementary file 1 [file sensors-25-03918-s001.zip › sensors-3622407-SI.pdf]

Distance Measurement and Data Analysis for Civil Aviation at 1000 Frames Per Second Using Single-Photon Detection Technology

In the application scenario of long-distance ranging, the ranging system has a maximum single-value measurement range, also known as the maximum unambiguous distance. This effect is referred to as Doppler ambiguity [31], and it is determined by the laser pulse period T. To ensure that the ranging system can perform single-value measurements, the laser pulse period T must satisfy the following condition:

$$T_{LD} \geq \frac{2R_{max}}{c} \tag{S1}$$

Here, R_{max} denotes the maximum unambiguous range, and c is the speed of light. As shown by the above equation, when the laser pulse repetition frequency is too low, the system cannot ensure unambiguous ranging, resulting in range ambiguity. The formation process of Doppler-induced range ambiguity is illustrated in Supplementary Figure S1(a).

From equation (S2), it can be seen that when the round-trip time of the laser pulse Δt equals the pulse period T_{LD}, the distance between the ranging system and the target object is the maximum unambiguous range R_{max}:

$$R_{max} = \frac{cT_{LD}}{2} \tag{S2}$$

f_{LD} denotes the frequency of the laser pulse. If Doppler-induced range ambiguity occurs during the measurement, the corresponding target distance R is given by:

$$R = \frac{c(mT_{LD}+t_{R_1})}{2},\ m = 0,1,2 .... \tag{S3}$$

As illustrated in Supplementary Fig. S1(b), this study employs a triple-frequency multiplexing strategy to determine the true distance R by resolving the integer ambiguity m in Equation (S3). In this scheme, laser pulse trains are cyclically emitted at repetition frequencies of $f_1 = 50kHz$, $f_2 = 49.5kHz$, and $f_3 = 49kHz$. Each frequency comprises 50 consecutive pulses—corresponding to approximately 1 ms of continuous emission—before switching to the next frequency in sequence.

The frequencies f_1, f_2, and f_3 are carefully chosen such that the number of pulses transmitted within the desired pulse repetition period $T_d = {}^1/_{f_{rd}}$ are pairwise coprime. This configuration guarantees that within the observation window, the echoes from the three pulse trains align at only one unique temporal offset, which corresponds to the true position of the target. The maximum unambiguous ranging distance achievable with this configuration is given by $R_{max} = \frac{c[T_1,T_2,T_3]}{2} = 300km$, where $[T_1, T_2, T_3]$ denotes the least common multiple of the period durations for the three repetition frequencies. The actual target distance can be resolved by solving the following system of equations [32]:

$$R_{real} = \frac{c(m_1T_1+t_1)}{2} = \frac{c(m_2T_2+t_2)}{2} = \frac{c(m_3T_3+t_3)}{2} \tag{4}$$

In Equation (S4), m_1, m_2, and m_3 denote the cycles associated with three ranging signals of different repetition frequencies and are the unknowns to be determined. T_1, T_2, and T_3 represent the corresponding pulse periods, known a priori. t_1, t_2, and t_3 indicate the bin positions of the ranging

peaks within the original sampled data. As indicated by the equation, the system necessitates integration of three consecutive single-frame measurements—each obtained at a distinct repetition frequency—to obtain a target range estimation.

In Figs.S1(a), the echo of pulse 1 represents the signal detected by the ranging system after a time delay associated with pulse 1. The echo of pulse 2 can be interpreted either as the echo generated by pulse 2 from the same target (i.e., at distance R_1), or alternatively, as the echo of pulse 1 reflected from a target located at distance R_2. The distance R_2 can be expressed as:

$$R_2 = \frac{c(mT_{LD}+t_{R_1})}{2},\ m = 0,1 \tag{S5}$$

The term t_{R_1} represents the time interval between the first pulse and its echo. It is evident that the echo of the second pulse induces Doppler blurring of the distance. Therefore, when the ranging system's frequency is sufficiently high and the required measurement distance is sufficiently large, Doppler blurring of the distance will occur. For single-valued ranging, the system must wait for a sufficiently long time after transmitting the pulse before sending the next pulse, ensuring the echo from the target is received before the emission of the next pulse.

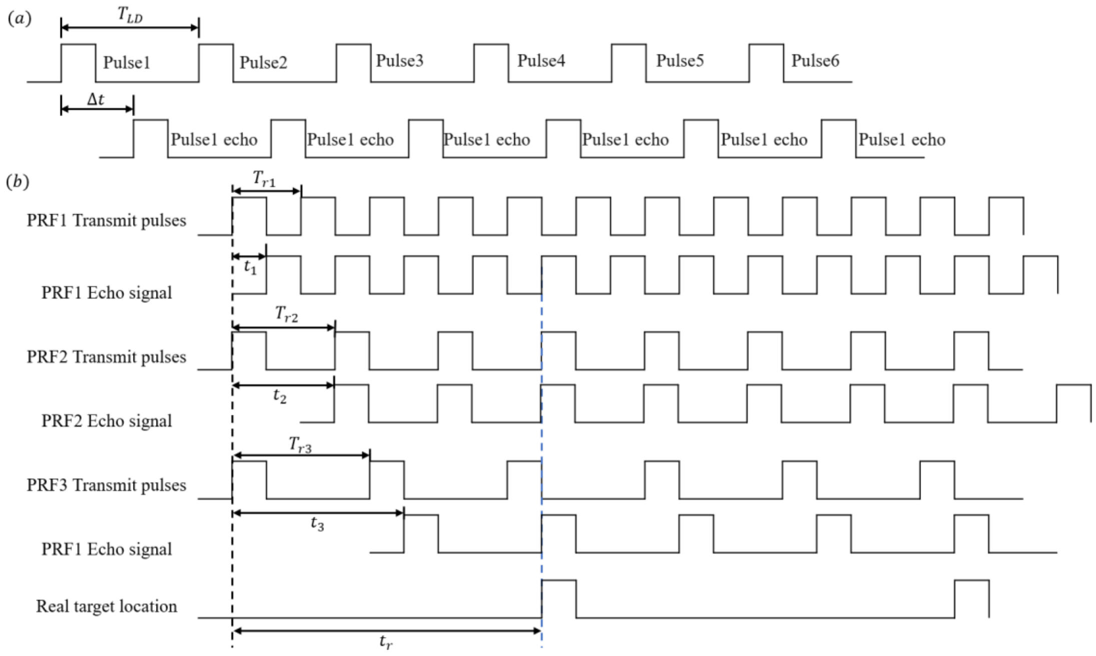

Figs.S1. (a) The process of the formation of blurred distances. (b) Principle of Multi-frequency Technology.

Figs.S2 illustrates the flowchart of the data denoising algorithm selected in this paper. Given that the raw data obtained from the distance measurement experiments exhibits a linear structure and there is a significant density difference between the signal and noise, a density-based clustering denoising algorithm has been chosen.

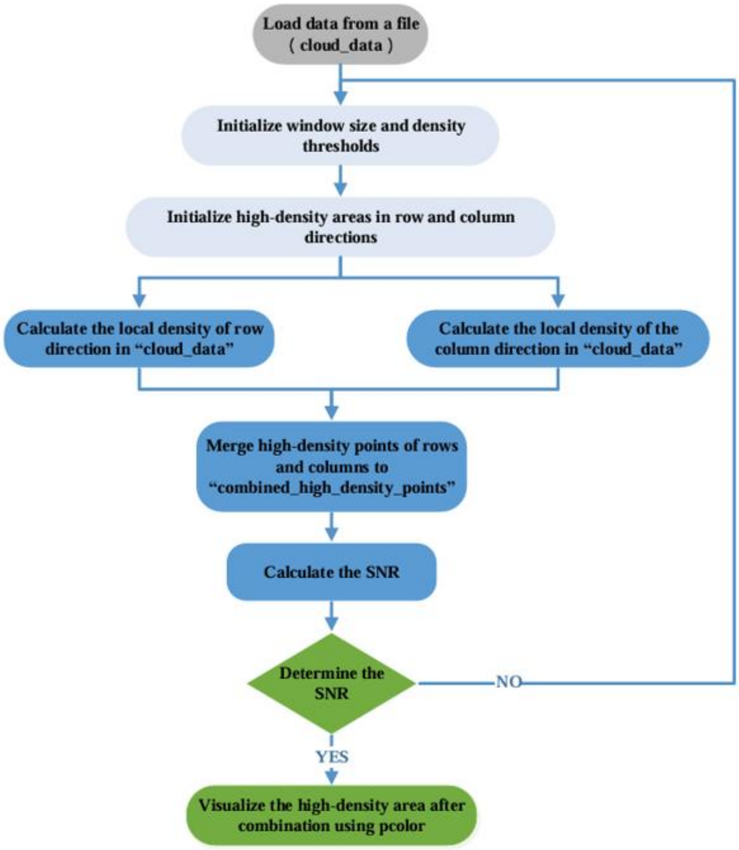

Figs.S2. Flowchart of the denoising algorithm based on density clustering.
